# Curvularia lunata Peritonitis in Continuous Ambulatory Peritoneal Dialysis: An Occupational Exposure at Play

**DOI:** 10.7759/cureus.96861

**Published:** 2025-11-14

**Authors:** Balamurugan Swaminathan, Manorajan Rajendran, Jegan Arunachalam, Padmakumar C, Mahesh Prabhu S

**Affiliations:** 1 Nephrology, Madurai Medical College, Madurai, IND; 2 Microbiology, Madurai Medical College, Madurai, IND

**Keywords:** continuous ambulatory peritoneal dialysis (capd), curvularia lunata, end-stage renal disease (esrd), fungal peritonitis, phaeohypomycosis

## Abstract

This is a case report of rare fungal peritonitis caused by the dematiaceous mold *Curvularia lunata* in a 45-year-old gentleman, a mason by occupation, who was a hypertensive and non-diabetic, chronic kidney disease (CKD) stage 5 patient undergoing continuous ambulatory peritoneal dialysis (CAPD). This represents one of the few documented cases globally notable for its occupational exposure (soil) as a likely risk factor. This patient presented with classic symptoms of peritonitis and blackish discolouration of the transfer set of the peritoneal dialysis (PD) catheter. Diagnosis was based on the microscopic identification of distinctive curved conidia with an enlarged hyperpigmented third cell - a hallmark feature differentiating it from other fungi of the same species. Successful management required urgent catheter removal and intravenous liposomal amphotericin B. This case underscores the life-threatening nature of fungal peritonitis (mortality: 20-45%) and establishes occupational soil exposure as a risk.

## Introduction

Peritoneal dialysis (PD) is an established modality of renal replacement therapy for patients with chronic kidney disease (CKD) and end-stage renal disease (ESRD); PD-associated peritonitis remains its most important complication and a leading cause of technique failure and hospitalization. Although most peritonitis episodes are bacterial, fungal peritonitis (FP) is an uncommon but clinically important entity, representing a small single-digit proportion of PD peritonitis episodes, and is associated with substantial morbidity, frequent catheter removal and transfer to hemodialysis, and variable but sometimes high mortality [1,2]. *Candida* species predominate among fungal isolates, but a heterogeneous group of moulds and non-*Candida* yeasts (including dematiaceous/phaeohyphomycotic fungi) have been reported in PD patients [2,3,4,5,6]. Phaeohyphomycosis peritonitis refers to infections caused by pigmented filamentous fungi that contain melanin in their cell walls, such as *Curvularia*, *Bipolaris*, *Exophiala*, and *Alternaria* species. These organisms are ubiquitous in soil and plant material and can behave as opportunistic pathogens, particularly in immunocompromised hosts or when indwelling catheters and breaches in peritoneal integrity provide a portal of entry. Their melanin content is thought to confer resistance to host immune responses and certain antifungal agents, contributing to the often protracted and relapsing course of infection. Recognized risk factors for FP include prior bacterial peritonitis, recent or prolonged broad-spectrum antibiotic exposure, immunosuppression, malnutrition, and breaches in exchange sterility; environmental or occupational exposures have also been implicated in isolated cases of unusual fungal pathogens [2,3]. The International Society for Peritoneal Dialysis (ISPD) recommends prompt recognition of FP and early catheter removal together with appropriate systemic antifungal therapy to reduce mortality and preserve patient safety [1]. *Curvularia lunata* is a soil-associated dematiaceous mould that only rarely causes human infection; isolated case reports document *C. lunata* peritonitis in PD patients, and occupational or environmental soil/plant exposure may be an important contextual clue in such cases, providing the rationale for reporting this case [3,4,5].

## Case presentation

**Figure 1:**
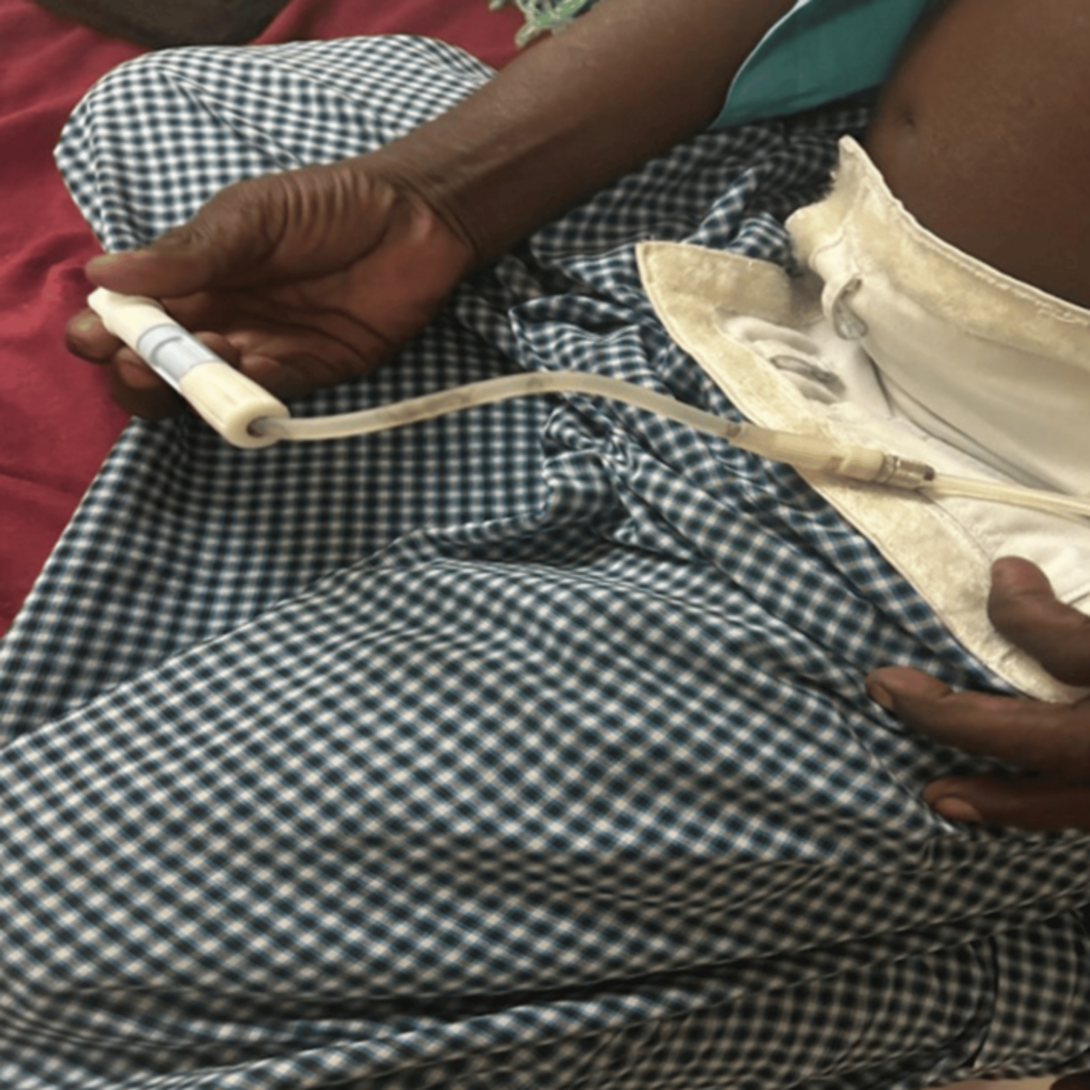
Transfer set showing a blackish discoloration of the continuous ambulatory peritoneal dialysis (CAPD) catheter

A 45-year-old construction worker with ESRD (hypertensive nephrosclerosis) and severe cardiomyopathy was initiated on continuous ambulatory peritoneal dialysis (CAPD) in January 2023. His occupation involved daily soil manipulation - a key risk factor overlooked in initial assessments. He was on a 2.5% dextrose solution, three exchanges per day, with adequate ultrafiltration. He had two episodes of bacterial peritonitis episodes on May 2023 and December 2023, which were treated with intraperitoneal antibiotics and recovered. He presented again in August 2024 with symptoms of peritonitis, cloudy dialysate fluid in the drain bag, and slow drain. On examination, he had jet-black discoloration of the transfer set tubing (Figure 1).

**Figure 2:**
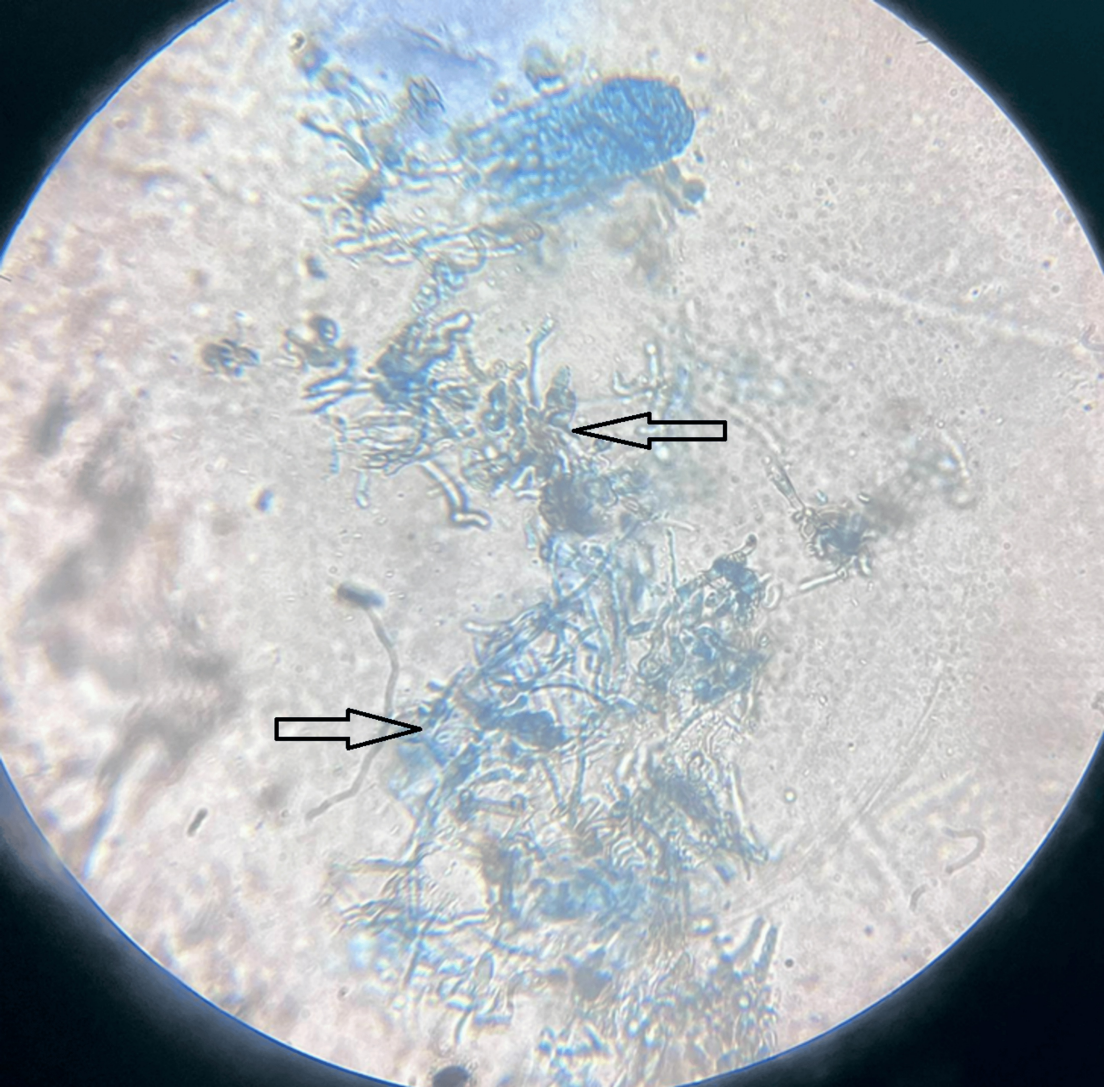
Potassium hydroxide (KOH) mount fungal hyphae seen

Suspecting fungal infection, his transfer set was removed and sent for potassium hydroxide (KOH) mount and fungal culture. Peritoneal fluid analysis revealed leukocytosis with neutrophilic predominance (white blood cell (WBC) 450/μL; 80% polymorphonuclear leukocytes (PMNs)), but Gram stain and bacterial cultures remained negative. Serum inflammatory markers were markedly elevated (C-reactive protein (CRP) 182 mg/L; procalcitonin 12.4 ng/mL). He was started on intraperitoneal antibiotics (vancomycin and amikacin). However, his transfer set scraping analysis in KOH mount showed fungal hyphae (Figure 2).

**Figure 3:**
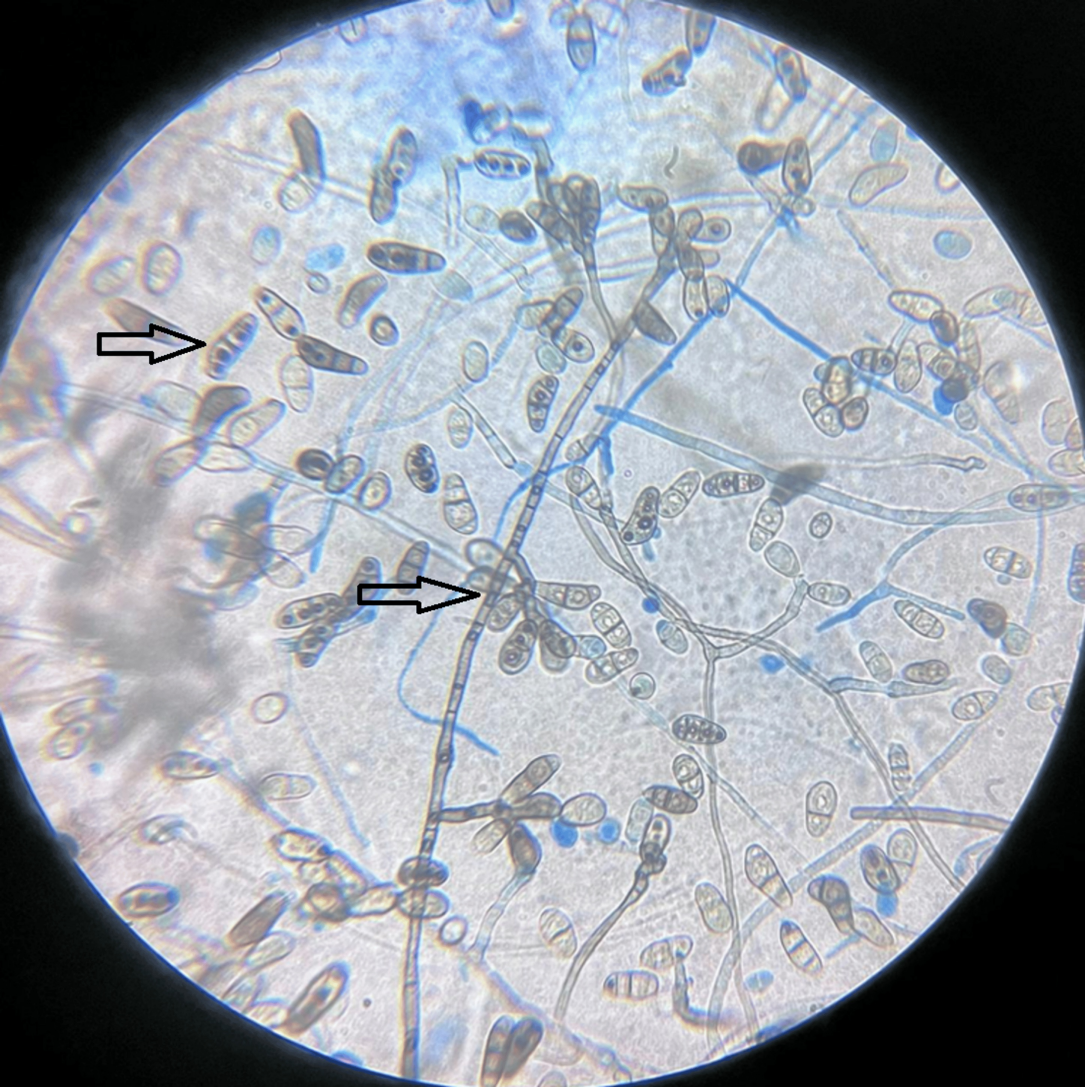
Lacto phenol cotton blue wet mount preparation - 40x magnification

He was started on anti-fungal fluconazole. Despite five days of therapy, the patient had a persistent fever and cloudy dialysate. Culture of transfer set after a week revealed growth of fungal species - *Curvularia lunata* (Figure 3).

**Figure 4:**
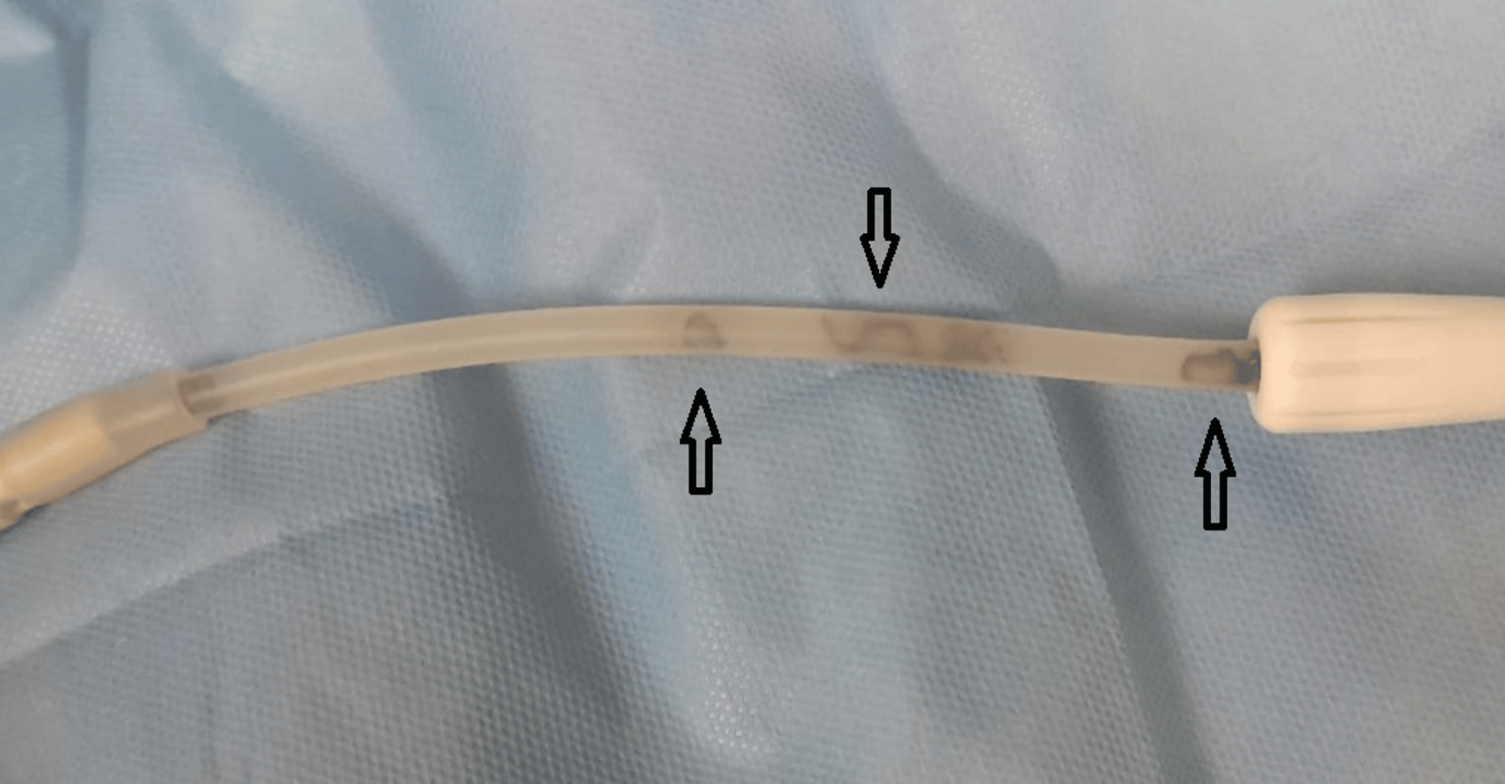
Removed continuous ambulatory peritoneal dialysis (CAPD) catheter showing black fungal hyphae

In view of fungal peritonitis, the patient's CAPD catheter was removed and sent for culture. The patient was started on IV liposomal amphotericin B 3-5 mg/kg to reach a cumulative dose of 2 g. The CAPD catheter that was removed also showed brown conidiophores with 3-4 mm diameter septate hyphae, resulting in a diagnosis of *C. lunata* PD peritonitis (Figure 4).

The patient was discharged in a clinically stable condition and shifted to maintenance hemodialysis.

## Discussion

*C. lunata* is a type of fungus (pheohypomycosis) that can occasionally cause infections in humans, particularly in immunocompromised individuals, such as those undergoing dialysis. It is part of the genus *Curvularia*, which is commonly found in soil [4], decaying plant matter, and various organic substrates. While *C. lunata* is more often associated with plant-related diseases or infections in agricultural settings, in humans, a wide spectrum of infections, including keratitis, cutaneous infections, sinusitis, allergic bronchopulmonary disease, pneumonia, chronic ambulatory peritoneal dialysis-related infections, endocarditis, and disseminated infections, have been described [7]. Indwelling devices, especially the presence of dialysis catheters, can serve as a direct entry point for fungi into the body. Contaminated dialysis equipment, like the use of non-sterile or improperly sterilized equipment and solutions during dialysis exchanges or procedures, can increase the risk of fungal infections. Usually, the collected sample is examined under a microscope using KOH (potassium hydroxide) preparation to look for fungal elements. In the case of *C. lunata*, characteristic features such as septate hyphae and curved conidia are observed. Giemsa stain or Gram stain may also help visualize fungal elements, but KOH and fungal stains are generally more specific. Fungal cultures: The sample is inoculated onto fungal culture media (e.g., Sabouraud dextrose agar). *Curvularia* species typically grows well at room temperature (25-30°C) and produces a characteristic appearance with dark, slow-growing colonies. The colonies of *Curvularia* species are woolly, initially white, then becoming grayish-black with black reverse [8]. The conidiophores are simple or branched, bent at points where conidia originate, and are hence called a sympodial geniculate growth. The conidia are 8-14 x 21-35 µm in size and have three septae, i.e., four cells, almost always curved at the third cell from the base, which is larger and darker than the others. The conidia are in an acropetal pattern and usually have a curved appearance, having broader points at the end. The cell in the middle of the curve is often larger than those toward the end. Additional molecular methods (such as PCR and DNA sequencing) can also be used to confirm the identity of *C. lunata*, especially in difficult cases where traditional culture and microscopy are not definitive. *C. lunata* can cause disseminated infections in immunocompromised patients, so detecting it in the blood can confirm that the infection has spread beyond the initial site. In some cases, tissue biopsy may be performed, particularly for sinus, lung, or skin infections. Antifungal therapy is the main treatment for *C. lunata* infections, with common drugs including fluconazole, voriconazole, or amphotericin B, depending on the severity and location of the infection.

Fungal peritonitis due to dematiaceous fungi such as *C. lunata* poses a unique therapeutic challenge because standardized antifungal susceptibility testing (AFST) protocols and clinical breakpoints for these organisms are not well established. In vitro susceptibility results often show variable responses to amphotericin B, azoles, and echinocandins, with some isolates demonstrating intrinsic or acquired resistance [9]. Varughese et al. reported a case of *C. lunata* peritonitis in a PD patient with amphotericin B resistance, underscoring the need for individualized antifungal therapy guided by laboratory testing where feasible [9]. Bibashi et al. also documented significant morbidity and catheter loss in a large series of fungal peritonitis cases, emphasizing the poor outcomes associated with delayed recognition and limited antifungal options [10].

Because dematiaceous fungi are environmental saprophytes, their presence in peritoneal dialysis effluent highlights the risk of medical device-associated infections through contamination of catheters, dialysate connections, or exchange surfaces. Adherence to strict aseptic technique, early suspicion of fungal etiology in refractory or relapsing peritonitis, and timely catheter removal remain critical to improving outcomes. Routine culture and antifungal susceptibility testing of such isolates, although not yet standardized, can aid epidemiological understanding, guide antifungal stewardship, and contribute to infection control in PD programs. [9,10]

In cases of peritoneal dialysis-associated peritonitis, antifungal agents may be given both intravenously and intraperitoneally. In case of fungal peritonitis, it is recommended that immediate catheter removal be performed when fungi are identified in PD effluent and treatment with an appropriate antifungal agent be continued for at least two weeks [2], and switching to hemodialysis might be considered. However, delayed treatment or recurrent infections can lead to serious complications, including sepsis, peritonitis, and even loss of peritoneal dialysis access. 

## Conclusions

This case report highlights the occurrence of fungal peritonitis caused by *C. lunata* in a CKD patient who worked in building construction, undergoing CAPD. Despite its relatively low incidence, fungal infections in peritoneal dialysis patients, especially those caused by *C. lunata*, should be considered in the differential diagnosis when a patient presents with repeated episodes of peritonitis, particularly after getting treated for bacterial peritonitis and with blackish discolouration of the transfer set as a clue to diagnosis. The infection in this case was diagnosed through microscopic examination and fungal culture of transfer set scrapings and capd catheter, which revealed the characteristic features of *C. lunata*, including septate hyphae and curved conidia. This underscores the importance of maintaining a high index of suspicion for fungal pathogens, particularly in immunocompromised patients on dialysis particularly whose occupation exposes them to soil. The case also emphasizes the need for careful attention to aseptic techniques during dialysis exchanges, proper training under supervision and the importance of early intervention in preventing the occurrence of fungal infections.
